# Hand grip strength is inversely associated with total daily insulin dose requirement in patients with type 2 diabetes mellitus: a cross-sectional study

**DOI:** 10.7717/peerj.15761

**Published:** 2023-07-20

**Authors:** Da-shuang Chen, Yun-qing Zhu, Wen-ji Ni, Yu-jiao Li, Guo-ping Yin, Zi-yue Shao, Jian Zhu

**Affiliations:** Department of Endocrinology, Nanjing First Hospital, Nanjing Medical University, Nanjing, Jiangsu, China

**Keywords:** Type 2 diabetes mellitus, Hand grip strength, Total daily insulin dose

## Abstract

**Background:**

Short-term (2 weeks to 3 months) insulin intensive therapy using continuous subcutaneous insulin infusion (CSII) can improve islet beta cell function and prolong glycemic remission in patients with newly diagnosed type 2 diabetes mellitus (T2DM). However, the total daily insulin dose (TDD, IU/kg/d) required to achieve near-normoglycemic control with CSII still needs to be frequently adjusted based on blood glucose monitoring. Although real-time continuous glucose monitoring (rtCGM), which measures the interstitial fluid glucose concentration continuously without much difficulty, facilitates the adjustment of insulin dosage, its adoption in the T2DM population is strictly limited by insurance coverage and lack of awareness of rtCGM among clinicians. Thus, it is of clinical significance to identify easy-to-use parameters that may allow a more rapid and accurate prediction of TDD requirement. This study aimed to explore the association between hand grip strength (HGS) and TDD requirement in patients with T2DM receiving CSII therapy.

**Methods:**

A total of 180 eligible patients with T2DM were enrolled in the study and divided into three groups based on their HGS: low (L), medium (M), and high (H). The TDD requirement was calculated on day 7 or 8 of CSII treatment. Anthropometric parameters, including HGS, skeletal muscle mass, skeletal muscle index (SMI) and 6-m gait speed, and laboratory data, were collected on the morning of the second day after admission, within the first 24 h of CSII therapy. These parameters were used to identify significant predictors of TDD requirement using Pearson or Spearman correlation test, and stepwise multiple regression analysis.

**Results:**

There were no significant differences in age, duration of T2DM, waist-to-hip ratio (WHR), body mass index (BMI), blood pressure, liver function, estimated glomerular filtration rate, triglyceride, total cholesterol, glycosylated hemoglobin A1c (HbA1c), homeostatic model assessment of insulin resistance (HOMA-IR), and homeostasis model assessment of beta cell function (HOMA-β) among the groups. The H group had higher body muscle mass-to-fat ratio (BMFR), skeletal muscle mass-to-fat ratio (SMFR), SMI, 6-m gait speed, and lower TDD requirement than the M and L groups. The HGS negatively correlated with TDD requirement (r = −0.33, *p* < 0.001) after adjusting for sex, age, BMI, WHR, HbA1c, Ln (HOMA-β), Ln (HOMA-IR), Ln (BMFR), Ln (SMFR), SMI, and 6-m gait speed. Multivariate stepwise regression analysis indicated that HGS was an independent predictor of TDD requirement in patients with T2DM (β = −0.45, *p* < 0 001).

**Conclusion:**

Lower HGS is associated with an increased TDD requirement in T2DM patients. HGS may facilitate the prediction of TDD requirement in T2DM patients receiving CSII therapy.

## Introduction

Diabetes mellitus is a complex and progressive metabolic disease that has reached epidemic proportions worldwide (Saeedi et al., 2019). Euglycemic control is essential for preventing acute and chronic diabetic complications (Lam et al., 2021). Although several types of antidiabetic medications are available, insulin is considered the most effective glucose-lowering agent (Taylor, Yazdi & Beitelshees, 2021). Furthermore, short‑term intensive insulin therapy using continuous subcutaneous insulin infusion (CSII) can improve islet beta cell function in patients with newly diagnosed type 2 diabetes mellitus (T2DM), thus prolonging glycemic remission (Weng et al., 2008; Weng, 2017; Kramer, Zinman & Retnakaran, 2013). Optimal CSII therapy is determined by accurately setting the total daily insulin dose (TDD) as well as the basal and bolus doses. Although TDD can be roughly calculated by multiplying body weight (kg) by 0.5–0.8 according to the guidelines of the insulin pump (Mu & Yin, 2012; Chun, Strong & Urquhart, 2019), it should be individualized because the degree of insulin sensitivity varies considerably among patients. Two small-scale studies found that factors such as sex, waist circumference, glucose control levels, and a more intricate parameter such as the ratio of the reduction in blood glucose per unit of insulin would benefit the prediction of optimal TDD requirement (Yang et al., 2019; Ma et al., 2016). Nevertheless, deriving individualized TDD requirement remains a challenging problem for clinicians. The TDD requirement still needs to be frequently adjusted based on blood glucose monitoring. Although real-time continuous glucose monitoring (rtCGM), which measures the interstitial fluid glucose concentration continuously without much difficulty, facilitates the adjustment of insulin dosage (Rodbard, 2017), its adoption in the T2DM population is strictly limited by insurance coverage and lack of awareness of rtCGM among clinicians. Therefore, it is necessary to identify more clinically easy-to-use and quantifiable factors to help physicians predict the TDD requirement.

Skeletal muscle is responsible for the largest portion of insulin-mediated whole-body glucose disposal, and insulin resistance in skeletal muscle plays a major role in the development of T2DM (Sylow et al., 2021). Hand grip strength (HGS) is a simple and cost-effective method for evaluating overall skeletal muscle strength and quality in clinical practice (Willems et al., 2017; Mearns, 2015; Balducci et al., 2014). There is evidence that HGS is associated with chronic diseases, such as diabetes, metabolic syndrome, and cardiovascular disease (Mearns, 2015; Li et al., 2018; Li et al., 2021; Lee et al., 2018), with higher relative HGS being associated with lower risks of diabetes and impaired fasting glucose. However, the association between HGS and TDD requirement has not been addressed in the literature. Therefore, we performed this cross-sectional study to evaluate the relationship between HGS and TDD requirement in T2DM patients receiving CSII therapy.

## Materials and Methods

### Study design

This was a retrospective, single-center, observational study. The Ethics Committee of Nanjing First Hospital, affiliated with Nanjing Medical University, approved this study and waived the requirement for written informed consent. All performed procedures were in accordance with the Declaration of Helsinki guidelines, including any relevant details. Three researchers extracted data of T2DM patients admitted to our department from the hospital database. The data analysis covered the period from Jun, 2019 to Dec, 2021. Inclusion criteria included the following: (1) newly diagnosed T2DM and glycosylated hemoglobin A1c (HbA1c) ≥8.0% on admission, or T2DM patients treated with two or more kinds of oral hypoglycemic agents for more than 3 months and HbA1c ≥7.5% on admission; (2) patient’s age ≥18 years and ≤70 years; (3) BMI between 18 and 35 kg/m^2^; (4) hypoglycemic regimen only consisting of CSII therapy during hospitalization; (5) reaching glycemic control (3.9 mmol/L < fasting capillary blood glucose <7.8 mmol/L, and 3.9 mmol/L< capillary blood glucose at 2 h after each of the three meals <11.1 mmol/L) after 4- or 5-day of treatment and its maintenance in the target range for the next three consecutive days. The exclusion criteria were similar to those used in the previous study (Zhu et al., 2019). Patients were excluded if they (1) were diagnosed with type one diabetes mellitus, latent autoimmune diabetes in adults, maturity-onset diabetes in youth, mitochondrial diabetes mellitus, or had a fasting C-peptide concentration <0.5 ng/mL; (2) had acute complications of diabetes on admission, such as diabetic ketoacidosis; (3) had severely impaired liver and kidney function, psychiatric disorders or infectious diseases; (4) had severe cardiovascular diseases, such as stroke, myocardial infarction or heart failure; (5) had cognitive diseases, alcoholism, cancers, or a history of drug abuse; (6) had symptomatic hypoglycemia episode or biochemical hypoglycemia from the sixth day to the eighth day of CSII therapy.

### Laboratory assessments

On the second day after admission, blood samples were drawn in a fasting state for biochemical tests. Serum insulin levels were measured using the Abbott Architect I2000 automated chemiluminescence immunoassay (Abbott Laboratories, Chicago, IL, USA). HbA1c levels were determined by high-performance liquid chromatography (Bio-Rad, Hercules, CA, USA). Homeostasis model assessment of insulin resistance (HOMA-IR), and homeostasis model assessment of beta cell function (HOMA-β) were calculated according to previously reported methods (Matthews et al., 1985).

### Insulin dosage calculation and blood glucose profiles

When the glycemic control of those enrolled subjects was reached after 4- or 5-day CSII treatment and kept in target range for next three consecutive days, namely, at the day 7 or day 8 during CSII therapy, the TDD requirement was calculated as follows: TDD (IU/kg/day) = daily total insulin dose/body weight. At the same date, the pre and 2h-postprandial blood glucose levels of each meal, as well as the prebedtime blood glucose, were collected and used for analysis of blood glucose profiles.

### Skeletal muscle mass and strength measurement

Body composition parameters, including skeletal muscle mass (SMM), soft lean mass (SLM), body fat mass (BFM), and skeletal muscle index (SMI), were tested using multifrequency BIA (InBody 770 body composition analyzer; InBody, Seoul, South Korea). In brief, within the first 24 h of CSII therapy, the assessment was performed on the morning before breakfast of second day after admission to avoid interference with food mass, and after voiding urine and excrement to avoid interference with weight measurement. Participants were asked to remove all accessories, socks, and jewelry before testing. SMI was calculated using the following formula: SMI = SMM of the upper and lower limbs (kg)/height (m)^2^. HGS was measured using a dynamometer (JAMAR Hand dynameter 5030JO; Sammons Preston, Bolingbrook, IL, USA). The maximum reading of at least two trials in a maximum-effort isometric contraction was obtained from each hand, and the average value (kg) between the right and left hands was used for subsequent analysis. Gait speed was measured using the shortest time in seconds to complete a walk along a straight line of 6 m. A warm-up period of <5 min was followed by two walks, and the shorter time was recorded.

### Statistical analysis

Data were analyzed using the SPSS PASW Statistics 22 package (SPSS Inc., Chicago, IL, USA) as described in our previous study (Zhu et al., 2019). All continuous data were tested for normality using the Kolmogorov-Smirnov test. Normally distributed parameters were expressed as mean ± standard deviation, and non-normally distributed parameters were expressed as medians with interquartile ranges. Parameters that did not follow a normal distribution were mathematically transformed to improve the symmetry for subsequent analyses. One-way ANOVA with least significant difference (LSD) or Tamhane’s T2 *post hoc* test, Kruskal-Wallis one-way ANOVA with Dunns multiple comparisons test, and chi-square test followed by pairwise multiple comparisons (Bonferroni method to adjust the *p*-value) were used to analyze the differences among groups for parametric, nonparametric continuous variables, and sex, respectively. The relationships between variables and TDD requirement were analyzed by Pearson or Spearman correlation test, and the controlling variables for partial correlation between HGS and TDD requirement were selected according to their clinical significance or variables with *p*-value <0.05, in bivariate correlation analysis with TDD requirement. Multiple linear regression analysis was performed in a stepwise manner to select suitable variables for the model. Statistical significance was defined as a two-sided *p*-value <0.05.

## Results

### Baseline characteristics of patients

In total, 206 inpatients with T2DM who met the inclusion criteria were recruited for this study. Twenty-six participants were excluded because of inadequate clinical data. Finally, 180 patients (113 men and 67 women, aged 52.36 ± 10.35 years, BMI 24.85 ± 3.02 kg/m^²^ and HbA1c 10.61 ± 1.73%) were enrolled and subdivided into three groups according to the tertiles of HGS: a low (L) group with HGS ≤ 24.20 kg, a medium (M) group with HGS 24.20–35.40 kg, and a high (H) group with HGS > 35.40 kg. As shown in Table 1, there were no significant differences in age, duration of T2DM, BMI, waist-to-hip ratio (WHR), blood pressure, liver function, estimated glomerular filtration rate, HbA1c, triglyceride, total cholesterol, LDL-cholesterol, HOMA-β, or HOMA-IR among all groups. The male-to-female ratio, SMI, body muscle mass-to-fat ratio (BMFR), and skeletal muscle mass-to-fat ratio (SMFR) showed a significant progressive increase as HGS increase, however, TDD significantly decreased as HGS increased in T2DM patients. Moreover, the H and M groups had lower HDL-cholesterol than L group, and the H group had a significantly increased 6-m gait speed compared with the L and M groups.

**Table 1:** The baseline characteristics of participants by categories of HGS levels.

	L group *n* = 60	M group *n* = 60	H group *n* = 60	*p* value
HGS (kg)	20.51 ± 2.46	30.30 ± 3.01^a^	40.97 ± 4.02^ab^	<0.001
Age (year)	54.92 ± 9.91	52.42 ± 10.33	50.95 ± 10.59	0.105
Sex (M/F)	9/51	47/13^a^	57/3^ab^	<0.001
Duration (month) of T2DM	2.00 (0.09–10.00)	1.00 (0.00–7.00)	1.50 (0.32–10.00)	0.591
Waist-to-hip ratio (WHR)	0.93 ± 0.05	0.94 ± 0.05	0.94 ± 0.05	0.410
BMI (kg/m^2^)	24.25 ± 2.51	24.90 ± 3.03	25.38 ± 3.38	0.120
Systolic BP (mmHg)	124.50 (117.20–132.00)	120.00 (117.50–134.50)	122.00 (116.80–140.50)	0.799
Diastolic BP (mmHg)	80.00 (70.00–80.50)	80.00 (76.00–90.00)	80.00 (76.25–90.00)	0.055
ALT (U/L)	23.10 (14.70–31.00)	19.70 (14.00–27.00)	24.40 (16.00–40.50)	0.141
AST (U/L)	16.00 (12.00–20.50)	15.25 (12.38–20.23)	17.40 (13.00–24.75)	0.183
eGFR (mL/min/1.73 m^2^)	103.41 ± 19.87	104.87 ± 13.97	104.76 ± 16.82	0.876
Triglyceride (mmol/L)	1.55 (1.21–2.29)	1.38 (1.13–2.23)	1.48 (0.97–2.67)	0.575
Total cholesterol (mmol/L)	5.15 ± 1.36	5.02 ± 1.12	5.04 ± 1.13	0.827
HDL-cholesterol (mmol/L)	1.25 ± 0.23	1.11 ± 0.25^a^	1.12 ± 0.26^a^	0.003
LDL-cholesterol (mmol/L)	2.70 ± 1.00	2.92 ± 0.93	2.70 ± 0.94	0.359
HbA1c (%)	10.59 ± 1.91	10.84 ± 1.83	10.40 ± 1.42	0.394
HOMA-IR	3.10 (1.73–4.49)	2.92 (1.86–4.64)	2.14 (1.71–3.31)	0.279
HOMA-β	25.94 (13.88–44.97)	28.87 (14.80–57.48)	20.31 (9.83–43.24)	0.078
6-m gait speed (m/s)	1.25 ± 0.14	1.29 ± 0.12	1.35 ± 0.18^ab^	0.001
SMI (kg/m^2^)	6.44 ± 0.72	7.42 ± 0.81^a^	7.92 ± 0.65^ab^	<0.001
BMFR	1.87 (1.59–2.21)	2.52 (2.03–3.01)^a^	2.81 (2.40–3.33)^ab^	<0.001
SMFR	1.07 (0.91–1.25)	1.48 (1.18–1.75)^a^	1.66 (1.39–1.98)^ab^	<0.001
TDD (IU/kg/d)	0.67 ± 0.20	0.54 ± 0.17^a^	0.46 ± 0.11^ab^	<0.001

**Note:**

HGS, hand grip strength, BMI, body mass index; BP, blood pressure; ALT, alanine aminotransferase; AST, aspartate aminotransferase; eGFR, estimated glomerular filtration rate; HbA1c, glycosylated hemoglobin A1c; HOMA-IR, homeostatic model assessment of insulin resistance; HOMA-β, homeostasis model assessment of pancreatic beta cell function; SMI, skeletal muscle index; BMFR, body muscle mass-to-fat ratio; SMFR, skeletal muscle mass-to-fat ratio; TDD, total daily insulin dose. a compared with the L Group (*p* < 0.05), b compared with M Group (*p* < 0.05). Data were presented as mean ± SD or median with interquartile range.

### Blood glucose profiles

There were no statistically significant differences in the blood glucose profiles among the three groups (Table 2).

**Table 2:** Blood glucose profiles of each group.

Group BG (mmol/L)	L group	M group	H group	*p* value
MBG	7.95 ± 1.07	7.82 ± 0.92	7.87 ± 1.11	0.780
Breakfast				
Premeal BG	6.20 ± 1.11	5.99 ± 0.85	6.27 ± 1.10	0.318
2hPG	9.53 ± 2.50	9.79 ± 2.32	9.52 ± 2.58	0.799
Lunch				
Premeal BG	7.48 ± 2.15	6.99 ± 1.76	7.52 ± 1.94	0.258
2hPG	8.31 ± 1.92	8.64 ± 2.00	8.25 ± 2.01	0.521
Dinner				
Premeal BG	7.70 ± 2.02	7.38 ± 1.81	7.03 ± 1.54	0.129
2hPG	8.66 ± 2.51	8.25 ± 1.70	8.62 ± 2.14	0.515
Prebedtime	7.72 ± 1.37	7.74 ± 1.54	7.86 ± 1.68	0.876

**Note:**

MBG, mean blood glucose; BG, blood glucose; 2hPG, 2-hour postprandial blood glucose.

### Correlation and regression analysis

The correlation test showed that sex, BMI, HbA1c, Ln (HOMA-IR), Ln (BMFR), Ln (SMFR), SMI, 6-m gait speed, and HGS were correlated with TDD requirement in all subjects, respectively (Table 3). Additionally, HGS still negatively correlated with TDD requirement after adjustment for sex, age, BMI, WHR, HbA1c, Ln (HOMA-β), Ln (HOMA-IR), Ln (BMFR), Ln (SMFR), SMI and 6-m gait speed (r = −0.33, *p* < 0.001). We further set the TDD requirement as a dependent variable, and all parameters except for TDD listed in Table 1 (parameters that did not fulfill a normal distribution were mathematically transformed) as independent variables, and performed a multiple linear stepwise regression analysis to assess the independent effects of different parameters on the TDD requirement. As a result, we obtained two models (Table 4). Meanwhile, we checked the degree of multicollinearity among all parameters included in the analysis, the eigenvalue >0, condition index <30, and variance inflation factor = 1.0 for two models, indicate that there was no collinearity problem. Our data showed that HGS emerged as an independent variable associated with TDD requirement and could predict 20.0% of the TDD requirement variance in T2DM patients (Table 4, Model 1). As shown in Model 2 (Table 4), HGS and HbA1c were independent predictors of TDD requirement.

**Table 3:** The correlation between TDD and different parameters.

Parameter	r	*p* value
Sex	0.35	<0.001
Age	−0.02	0.797
BMI	−0.18	0.014
WHR	−0.03	0.731
Ln (duration of T2DM)	0.09	0.299
Ln (triglyceride)	0.11	0.156
HbA1c	0.37	<0.001
Ln (HOMA-β)	0.04	0.589
Ln (HOMA-IR)	0.24	0.002
Ln (BMFR)	−0.19	0.012
Ln (SMFR)	−0.16	0.037
SMI	−0.40	<0.001
6-m gait speed	−0.21	0.005
HGS	−0.47	<0.001
HGS*	−0.33	<0.001

**Notes:**

TDD, total daily insulin dose; Ln, base-e logarithm; BMI, body mass index; WHR, waist-to-hip ratio; HbA1c, glycosylated hemoglobin A1c; HOMA-β, homeostasis model assessment for beta cell function; HOMA-IR, homeostatic model assessment for insulin resistance; BMFR, body muscle mass-to-fat ratio; SMFR, skeletal muscle mass to-fat ratio; SMI, skeletal muscle index.

*After adjustment for sex, age, BMI, WHR, HbA1c, Ln(HOMA-β), Ln(HOMA-IR), Ln(BMFR), Ln(SMFR), SMI, and 6-m gait speed.

**Table 4:** Stepwise multiple linear regression analysis with TDD as the dependent variable.

	Adjusted *R*^2^	Unstandardized β	β Std. error	Standardized β	t	*p* value
Model 1	0.20					<0.001
Constant		0.79	0.05		16.17	<0.001
HGS		−0.01	0.00	−0.45	−5.49	<0.001
Model 2	0.34					<0.001
Constant		0.44	0.09		5.18	<0.001
HGS		−0.01	0.00	−0.47	−6.30	<0.001
HbA1c		0.04	0.01	0.37	4.98	<0.001

**Note:**

TDD, total daily insulin dose; HGS, hand grip strength; HbA1c, glycosylated hemoglobin A1c.

### Discussion

To the best of our knowledge, this is the first study to investigate the correlation between HGS and TDD requirement in T2DM patients receiving CSII therapy. We revealed a novel observation that HGS is negatively correlated with TDD requirement in T2DM patients. This association was independent of sex, age, BMI, WHR, HbA1c, HOMA-β, HOMA-IR, BMFR, SMFR, SMI, and 6-m gait speed, furthermore, the multivariate stepwise regression analysis indicated that HGS was an independent predictor of TDD requirement in T2DM patients. HGS may allow a more rapid and accurate prediction of TDD requirement for T2DM patients receiving CSII therapy.

Insulin therapy is most effective when dose titrations are performed regularly and frequently (Bergenstal et al., 2019). Unfortunately, previous clinical trials assessing effective insulin therapy in T2DM management based on TDD requirement have demonstrated that most patients using insulin are underdosed (Mungreiphy et al., 2015). Additionally, clinical evidence has shown that insulin titration is often postponed for a longer period due to a shortage of healthcare provider resources and clinical inertia in the insulin treatment of patients (Chun, Strong & Urquhart, 2019). Although this study only investigated inpatients, CSII therapy can be extended to ambulatory care for T2DM patients. More specifically, it is of practical significance to set an appropriate TDD to fit the real-world/outpatient setting, where we need to reduce the frequency of titration for individuals with diabetes.

Partially consistent with a previous study that found that waist circumference, baseline fasting blood glucose, and HbA1c were independently associated with TDD requirement (Yang et al., 2019), this study demonstrated that HGS and HbA1c were two independent predictors of TDD requirement. HGS has been used in numerous clinical studies to examine the association between skeletal muscle and T2DM (Balducci et al., 2014; Lee et al., 2018; Hamasaki, 2021). There has been significant evidence from epidemiological and observational studies that lower HGS is associated with higher rates of T2DM in the general population (Li et al., 2021; Lee et al., 2018; Kunutsor, Voutilainen & Laukkanen, 2020). HGS is negatively correlated with HOMA-IR and 2-h glucose levels in glucose-tolerant individuals (Li et al., 2018), especially children and adolescents (Jiménez-Pavón et al., 2012; Demmer et al., 2016; Benson, Torode & Singh, 2006). Alternatively, increased glucose levels were associated with impaired muscle quality, strength, and physical performance (Yoon et al., 2016; Leenders et al., 2013; Kalyani et al., 2015). Another study collected daily glucose level data from T2DM patients who were monitored at five time points per day (05:00 AM, before breakfast, 2 h after breakfast, before lunch, and before dinner) on 8 days during a 2-month period, and found that T2DM patients with low HGS displayed high glucose levels at 2 h after breakfast, before lunch, and dinner (Ogama et al., 2019). This study also confirms the validity of our findings, indicating that T2DM patients with low HGS may require more TDD than those with high HGS to achieve near-normoglycemic control.

In a common sense, TDD requirement can be recognized as an indirect index for insulin resistance, and it is reasonable that HOMA-IR should be a predictor of TDD requirement. In Pearson correlation analysis, Ln (HOMA-IR) was positively correlated with TDD requirement, however, the multiple linear stepwise regression analysis excluded Ln (HOMA-IR) from the models. There is a possibility that the validity of HOMA-IR as an indicator of insulin resistance varies significantly among patients with different BMI, beta cell function, and fasting glucose levels (Kang et al., 2005). A more crucial factor is that HOMA-IR, which is based on a patient’s fasting plasma insulin and glucose concentrations, primarily reflects hepatic insulin resistance rather than skeletal muscle insulin resistance (Matthews et al., 1985; Katsuki et al., 2001; Bonora et al., 2000). The skeletal muscle is the primary site of insulin-stimulated glucose disposal, accounting for as much as about 80% of an ingested glucose load (DeFronzo & Tripathy, 2009; Bouzakri, Koistinen & Zierath, 2005). Previous studies have demonstrated that patients have multiple muscular defects from the early stages of T2DM, including reduced muscle strength, mass, and endurance due to alterations in the electrical properties of the muscle membrane, mitochondrial function and morphology, and increased glycation of skeletal muscle myosin (Balducci et al., 2014; Orlando et al., 2016; Sayer et al., 2005). The decrease in the quantity and quality of skeletal muscle during the course of T2DM development significantly elevates insulin resistance of the whole body (Abdul-Ghani & DeFronzo, 2010), resulting in an increased TDD requirement in T2DM patients receiving insulin treatment. Based on the aforementioned studies (Balducci et al., 2014; Li et al., 2021; Hamasaki, 2021; Kunutsor, Voutilainen & Laukkanen, 2020) and our findings, HGS as an indicator of muscle functional capacity may facilitate the prediction of TDD requirement in patients with T2DM receiving CSII therapy.

However, the current study had some limitations. First, HGS was significantly lower in women than in men. As the sex distributions of the patient groups in this study were difficult to match, some bias due to sex mismatch could have been introduced as a result. Second, we did not use the glucose clamp technique to accurately assess the insulin sensitivity (M value) of the participants and did not include the M value in the statistical analysis, which could have affected the impact of the precise insulin resistance index on the TDD requirement prediction. Finally, further extensive studies are required to develop a formula using HGS and other parameters to precisely estimate TDD requirement.

## Conclusions

In conclusion, lower HGS was associated with increased TDD requirement in T2DM patients. HGS may be a practical parameter to help clinicians predict TDD requirement for T2DM patients receiving CSII therapy.

## Supplemental Information

10.7717/peerj.15761/supp-1 Supplemental Information 1 Raw data.

10.7717/peerj.15761/supp-2 Supplemental Information 2 Codebook.

## Additional Information and Declarations

### Data Availability

The following information was supplied regarding data availability: The raw data are available in the Supplemental Files.
