# Supplementary material for: Hand grip strength is inversely associated with total daily insulin dose requirement in patients with type 2 diabetes mellitus: a cross-sectional study
Source: PeerJ. 2023 Jul 20;11:e15761. doi: 10.7717/peerj.15761 (PMC10363338; doi:10.7717/peerj.15761)
Supplement: Supplemental Information 2 [file peerj-11-15761-s002.docx]

**Code book**

sex: 1= Male, 2= Female

HGS group

≤≤24.20 kg 1

24.20-35.40 kg 2

＞35.40 kg 3
